# Impact of PDGF‐BB on cellular distribution and extracellular matrix in the healing rabbit Achilles tendon three weeks post‐operation

**DOI:** 10.1002/2211-5463.12736

**Published:** 2020-02-05

**Authors:** Gabriella Meier Bürgisser, Olivera Evrova, Maurizio Calcagni, Chiara Scalera, Pietro Giovanoli, Johanna Buschmann

**Affiliations:** ^1^ Division of Plastic Surgery and Hand Surgery University Hospital Zurich Switzerland; ^2^ Laboratory of Applied Mechanobiology ETH Zürich Switzerland; ^3^ ab medica Cerro Maggiore Italy

**Keywords:** alpha‐SMA, collagen, PDGF‐BB, proteoglycan, rabbit Achilles tendon

## Abstract

Current methods for tendon rupture repair suffer from two main drawbacks: insufficient strength and adhesion formation, which lead to rerupture and impaired gliding. A novel polymer tube may help to overcome these problems by allowing growth factor delivery to the wound site and adhesion reduction, and by acting as a physical barrier to the surrounding tissue. In this study, we used a bilayered DegraPol^®^ tube to deliver PDGF‐BB to the wound site in a full‐transection rabbit Achilles tendon model. We then performed histological and immunohistochemical analysis at 3 weeks postoperation. Sustained delivery of PDGF‐BB to the healing Achilles tendon led to a significantly more homogenous cell distribution within the healing tissue. Lower cell densities next to the implant material were determined for +PDGF‐BB samples compared to −PDGF‐BB. PDGF‐BB application increased proteoglycan content and reduced alpha‐SMA^+^ areas, clusters of different sizes, mainly vessels. Finally, PDGF‐BB reduced collagens I and III in the extracellular matrix. The sustained delivery of PDGF‐BB via an electrospun DegraPol^®^ tube accelerated tendon wound healing by causing a more uniform cell distribution with higher proteoglycan content and less fibrotic tissue. Moreover, the application of this growth factor reduced collagen III and alpha‐SMA, indicating a faster and less fibrotic tendon healing.

## Graphical abstract

Sustained PDGF‐BB delivery via DegraPol^®^ implant to a fully transected rabbit Achilles tendon leads to a more homogenous cellular distribution in the healing tendon tissue *in vivo*. Increased proteoglycan content and reduced collagen III and alpha‐SMA^+^ areas confirm the positive effect of PDGF‐BB.

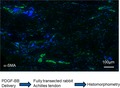

**Abbreviations**
Alpha‐SMA: alpha‐smooth muscle actin
DP: DegraPol^®^
ECM: extracellular matrix
En: endotenon
H&E: haematoxylin–eosin
i.v.: intravenous
PDGF‐BB: platelet‐derived growth factor‐BB
RZ: reactive zone
SPF: specific pathogen free
Z: zone

The healing of lacerated tendons is compromised by two main problems: rerupture and adhesion formation 1. An insufficiently strong scar tissue lacking the aligned collagen organization seen in normal healthy tendons often leads to reruptures. The second drawback, adhesion formation between the regenerating tendon and the surrounding tissue, occurs in 7–15% of all cases, leading to a reduced range of motion, which ends up in higher work disability and costs 2, 3. To address these two problems, an elastic biodegradable polymer tube was developed in our research team 4. It was tested for biocompatibility *in vivo* 4. The polymer used was DegraPol^®^ (DP) 5. Furthermore, we synthesized a new more elastic polymer to make a reversible expandable DP tube, which is surgeon‐friendly and easily applicable in a clinical setting 6. The performance of the electrospun tube acting as a physical barrier resulted in a 20% reduction in adhesion formation when applied in a full‐transection rabbit Achilles tendon model 7. Further development of the tube consisted in the incorporation of a growth factor, PDGF‐BB, into the electrospun mesh by spinning an emulsion 8. PDGF‐BB enhances tendon healing with respect to accelerated mitogenesis, chemotaxis and vascularization 9.

In order to get insight into the effects of the released PDGF‐BB from the electrospun tube implanted around a full‐transection rabbit Achilles tendon 10, we investigated the local distribution of tendon cells directly near the PDGF‐BB delivery device, in proximity and further away from it. Such information may provide deeper insights into how migration of tendon cells to the healing tendon is influenced by this growth factor. Moreover, proteoglycan content to get information about the tendon’s affinity for adhesion 11 and alpha‐SMA as a biomarker for fibrosis and scar formation 12 were determined. Finally, collagen I and III expression 13 was assessed to get insight into the state of healing progression.

Hence, the hypotheses of this histomorphometric study were as follows:

- PDGF‐BB influences the total cell density and the cellular distribution in the healing tendon,
- PDGF‐BB increases the proteoglycan content in the healing tendon,
- PDGF‐BB reduces scar formation, and
- PDGF‐BB accelerates the healing process.

## Materials and methods

### PDGF‐BB‐releasing DegraPol^®^ tubes

Emulsion electrospun DP tubes with incorporated PDGF‐BB were fabricated as reported previously 8. In addition, DP tubes made by coaxial electrospinning to produce hollow fibres loaded with PDGF‐BB 14 were produced as follows. Briefly, 10 wt % DP was used as a shell polymer solution, while 30 wt % polyethylene glycol (PEG) solution with PDGF‐BB was used as a core polymer solution. The polymer solutions for the shell and core were delivered through two syringes mounted on two different syringe pumps (SP210cZ and Aladdin‐1000; WPI, Friedberg, Germany), and different flow rates were used for each solution (shell: 1.5 mL·h^−1^; core: 0.5 mL·h^−1^). Voltage was 17 kV. The working distance between the spinneret and the collector was 18 cm. Electrospinning was performed at room temperature (22–24 °C) and less than 35% humidity. Analogously fabricated tubes without PDGF‐BB were used as controls.

### Animals

For this *in vivo* study, 12 female New Zealand White rabbits aged 12–16 weeks were used (Charles River, Research Models and Services, Sulzfeld, Germany). They were specific‐pathogen‐free (SPF). All animals were housed in pairs in two interconnected cages, each of them with a bottom area of 70 cm × 70 cm and a height of 62 cm (Indulab, Gams, Switzerland). The animals were maintained under controlled conditions: temperature 22 ± 1 °C, 45% relative humidity, 15 air changes per hour and a light/dark rhythm of 12 h. The rabbits had free access to water (automatic water supply), autoclaved hay and straw *ad libitum* and to standard pellet diet (Kliba Nafag, Nr. 3410, Provimi Kliba AG, Kaiseraugst, Switzerland). Ethical approval for the experiments was obtained from the veterinary office of Zurich, Switzerland (reference numbers 92/2009 and 193/2012). Prior to surgery, all animals were acclimatized to their environment for 2 weeks.

### Achilles tendon repair

The rabbits received premedication with 65 mg·kg^−1^ body weight ketamine and 4 mg·kg^−1^ xylazine 10. A venous catheter was inserted in the marginal ear vein. The rabbits were intubated with propofol i.v. 0.6–1.3 mg·kg^−1^. Anaesthesia was maintained with 1–2% isoflurane. In order to ensure systemic analgesia during the time of operation, 0.2–0.3 mg·kg^−1^ body weight butorphanol (Dr. E. Graeub AG, Berne, Switzerland) was applied preoperatively. The hind legs were shaved and cleaned with iodine (B. Braun Medical AG, Sempach, Switzerland). The Achilles tendon exposure was obtained through a paratendineal incision of cutis, subcutis and fascia. The medial M. gastrocnemius and lateral M. gastrocnemius of the Achilles tendon complex were then sliced perpendicularly to the length of the tendon 2 cm above the calcaneus, and one of the two fringed tendon stumps was sutured, while the fibre (USP 4.0 polypropylene) was then pulled through the DP tube (either with or without PDGF‐BB loading), before the second tendon stump was sutured. The fibre was knotted in order to minimize the gap between the stumps. The DP tube was then flipped over the wound. Subsequently, the wound was closed with a running suture (using a USP 6.0 polypropylene fibre) of the fascia and interrupted skin. Immediately postsurgery, a Durogesic Matrix Patch (Janssen‐Cilag AG, Schaffhausen, Switzerland) was applied with 4.2 mg fentanyl per patch in order to provide analgesia for about 72 h with 25 µg·h^−1^ fentanyl. Postoperative treatment included a cast having an angle of 180° at the ankle. The cast was well padded. Great attention was paid to make the casts not too tight so that it was tolerated well by the rabbits (they did not bite the cast). Three weeks postsurgery, the rabbits were euthanized in deep anaesthesia (100 mg·kg^−1^ ketamine and 4 mg·kg^−1^ xylazine) with 80 mg·kg^−1^ pentobarbital (Esconarkon *ad us. vet*., Streuli Pharma AG, Uznach, Switzerland) and the tendons were extracted and stored at −20 °C.

### Treatment groups

The 12 rabbits were randomly distributed into four groups with *n* = 3 in each group. All were operated on one hind leg 15. Of the operated legs, half were treated with an emulsion electrospun DegraPol^®^ tube and half were treated with a coaxially electrospun DegraPol^®^ tube, both with PDGF‐BB incorporated in one layer, facing the tendon; the other half were also treated with the two differently electrospun tubes, but without PDGF‐BB. The counter hind legs of all operated animals were not treated (NT) and served as control. For histological readouts, all Achilles tendons receiving a tube with PDGF‐BB (merged three emulsion and three coaxial, *n* = 6) and all tendons receiving a tube without PDGF‐BB (*n* = 6) were grouped. Moreover, all NT tendons were merged (*n* = 12).

### Histological assessments

After being thawed to room temperature, the tendons were cut at the repaired site (perpendicular to the Achilles tendon). Longitudinal sections were taken (towards bone and towards muscle) and halved for different embedding procedures in a sagittal plane. The pieces for paraffin‐embedding procedure were fixed in formalin for 24 h, then dehydrated, paraffin‐embedded and sectioned into 5‐µm‐thick slices. After deparaffinizing with xylene and rehydrating the sections, they were stained with haematoxylin–eosin (H&E), haemalaun–Sudan to stain the DegraPol^®^ polymer reddish 16 and Alcian blue according to the commonly established procedures.

H&E‐stained paraffin sections were used to evaluate total cell density in five different zones (Z): in the endotenon (Z2), in a reference zone near the endotenon (Z3 En), in the zone directly adjacent to the DegraPol^®^ (reactive zone Z4) and in a reference zone near the reactive zone (Z3 RZ). In addition, to compare to most preferably healthy tissue, cell densities were also assessed in the core of the tendon (Z10), which was similar to native tendon tissue (not treated = NT). Alcian blue‐stained paraffin sections were used to check whether sulfated proteoglycans were present. Zones were specified and numbered in accordance with a previous study 4.

The tendon pieces for cryogenic embedding were embedded in Tissue‐Tek^®^ O.C.T. (Sakura, Alphen aan den Rijn, The Netherlands, Europe), then frozen and cryosectioned into 5‐µm‐thick slices. After thawing, the sections were fixed with 4% paraformaldehyde for 10 min, then washed with 1×TBS and stained with H&E standard procedure.

Dense tenocyte area ratios were determined in cryogenic section based on the definition that they had to lie between DegraPol^®^ layers and be in the healing area; the dominating semiquantitative scores for the selected FOV were then weighted in terms of how much (%) it is represented in the FOV, and the ratio was defined as:

(1) $$\text{Ratio}=\frac{\text{score}\;\times \;\%}{100}.$$

For immunohistochemistry, paraffin‐embedded sections were deparaffinized with xylene and rehydrated, followed by an antigen retrieval step in 10 mm citrate buffer (pH 6.0) with 0.05% Tween‐20 for 20 min at 95 °C. If needed, depending on the epitope to stain, sections were permeabilized with 0.5% Triton X‐100 in 1×TBS for 10 min and subsequently washed three times with 1×TBS. Next, sections were blocked in 5% donkey serum and 1% BSA in 1×TBS for 1 h at room temperature. Afterwards, sections were incubated with mouse anti‐collagen I antibody (ab90395; Abcam, Lucerne, Siwtzerland, 1 : 200 dilution) or mouse anti‐collagen III antibody (AF5810; Acris, Wettingen, Switzerland, 1 : 200 dilution) or mouse anti‐alpha‐SMA antibody (A2547; Sigma‐Aldrich, Buchs, Switzerland, 1 : 500 dilution) diluted in 3% BSA in 1×TBS overnight at 4 °C.

For one marker, fluorescent immunohistochemistry was performed (alpha‐SMA), while for the others, chromogenic immunohistochemistry was performed (collagens I and III). For fluorescent immunohistochemistry, primary antibody solution was removed and samples were washed with 1×TBS before incubation with secondary donkey anti‐mouse Alexa‐488 antibody (A‐21202; Invitrogen, Basel, Switzerland, 1 : 500 dilution) and 10 µg·mL^−1^ 4′6‐diamidino‐2‐phenylindole dilactate (DAPI) (Sigma‐Aldrich) diluted in 3% BSA in 1×TBS for 1 h at room temperature. Afterwards, slides were washed in 1×TBS and mounted using Dako Fluorescence Mounting Medium (Agilent, Basel, Switzerland).

For chromogenic immunohistochemistry, the secondary antibody detection was performed using a biotinylated anti‐mouse IgG secondary antibody and streptavidin–horseradish peroxidase (HRP) (ZytoChem Plus HRP Kit Mouse; Zytomed Systems, Muttenz, Switzerland), followed by colorimetric detection using DAB substrate (DAB Substrate Kit High Contrast; Zytomed Systems) according to the manufacturer’s protocol. Afterwards, slides were washed in tap water and mounted using Faramount Aqueous Mounting Medium (Agilent).

Whole tissue sections were imaged on an automated slide scanner (Pannoramic 250 Flash II; 3Dhistech, Budapest, Hungary) or photographed with a Leica 6000 light microscope (Leica, Basel, Switzerland). For quantitative analysis, cell nuclei were counted in selected fields of view (FOVs) (*n* = 5). Semiquantitative analysis was performed for proteoglycans, dense tenocyte area and alpha‐SMA^+^ cells in selected FOVs (*n* = 5–15), examining the tissue quality with a previously established scoring scale (with 5 steps). For alpha‐SMA intensity scores, each FOV was subdivided into eight rectangles, totally 1116 score counts in 145 FOVs, in 9–12 tendons per treatment group. From all evaluated values, each third value was used in order to avoid statistical overpowering. Structures like vessels were indexed by intensity and size and were counted in a predefined area. Definition: Small full clusters with circumference < 100 µm; precursor vessels < 100 µm; precursor vessels > 100 µm; well‐developed vessels < 100 µm; well‐developed vessels > 100 µm. For alpha‐SMA‐cluster density, a total of 710 cluster counts in 145 FOVs were analysed.

### Statistical analysis

Data were analysed with statview 5.0.1 (BrainPower Inc, Cary, NC, USA). One‐way ANOVA was conducted. Pairwise comparison probabilities (*P*) were calculated using the Bonferroni *post hoc* test. *P *values < 0.05 were considered significant (*). If *P* < 0.01, this was marked by **, and if *P* < 0.001 by ***. Values were expressed as means ± standard deviations if not otherwise stated.

## Results

### Total cell density in different zones

**Figure 1:**
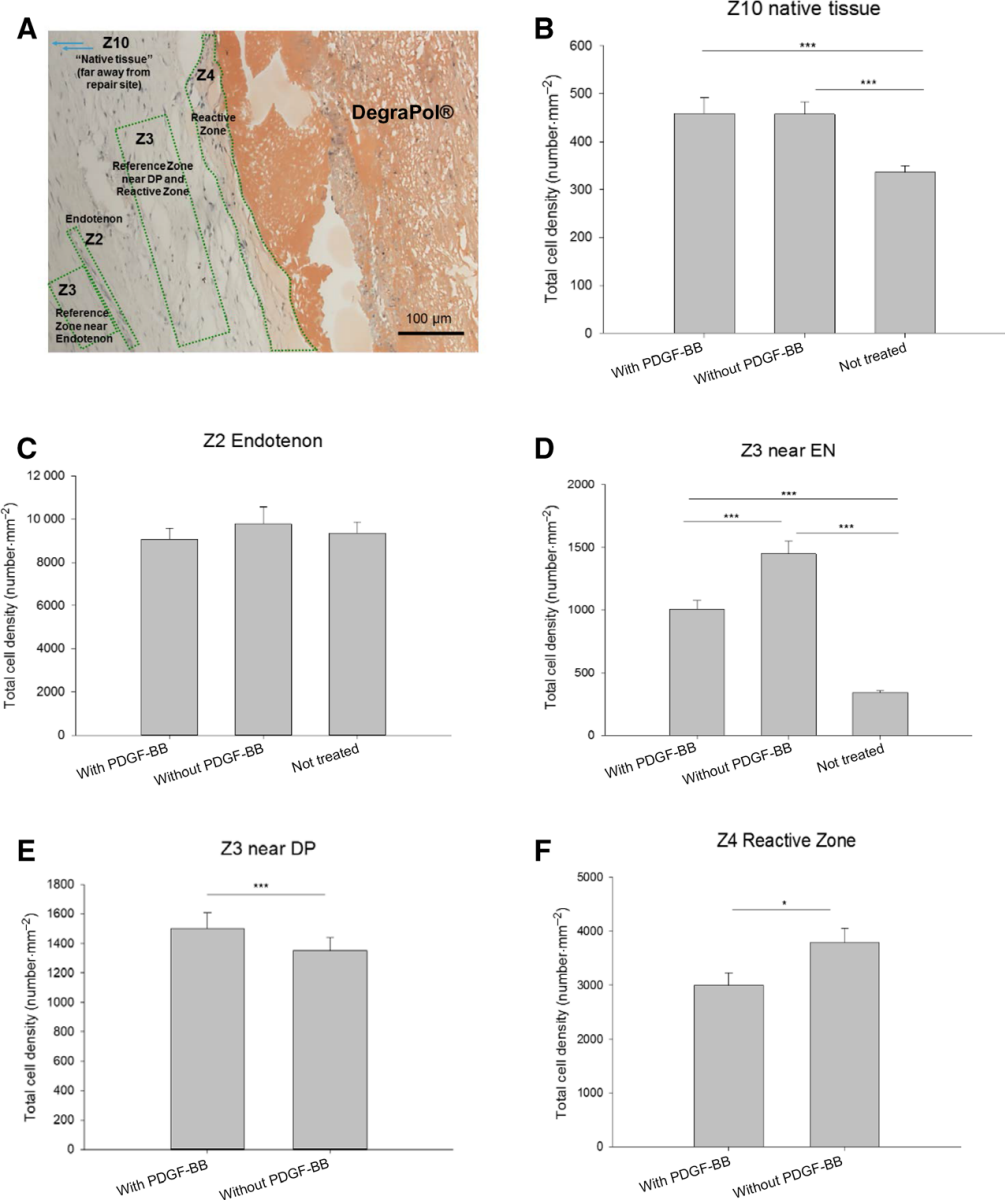
Total cell densities in different zones 3 weeks postsurgery. Overview of zones in a haemalaun–Sudan‐stained section, length of scale bar = 100 µm (A); total cell density in Z10 native core tissue (B); Z2 cells in the endotenon (C); zone Z3 lying near an endotenon (D); Z3 near DP (E); and Z4 reactive zone next to DP (F). Values are given as means ± standard error. The numbers for the zones (Z2, Z3, Z4 and Z10, respectively), were used in accordance with previously published work 4. One‐way ANOVA was performed. *P *values < 0.05 were considered significant (*). If *P* < 0.01, this was marked by **, and if *P* < 0.001 by ***. Error bars indicate standard deviations. Biological independent replicates *n* = 6. An overview of the whole longitudinal section is provided in Fig. S1.

After extraction, treated tendon specimen was longer and had a higher cross‐sectional area than NTs (Fig. S2). Analysis of total cellular distribution (Fig. 1) revealed similar cell densities in the native tendon tissue for both tendons (Z10; Fig. 1A and Fig. S1), treated with DP tubes loaded with or without PDGF‐BB; both, however, had significantly higher cell densities than the contralateral not treated tendons (Fig. 1B). While in the endotenon similar cell densities were observed (Fig. 1C), significantly lower cell densities in PDGF‐BB‐treated tendons were found in locations adjacent to the endotenon, here denoted as Z3 near endotenon (Fig. 1D) and next to the implant material (Fig. 1F) in the reactive zone Z4. These lower cell densities were at least partially compensated by higher cell densities close to DP in zone Z3 near DP (Fig. 1E), covering wider areas than the zones Z3 near endotenon and Z4 reactive zone, respectively.

### Dense tenocyte area ratio

**Figure 2:**
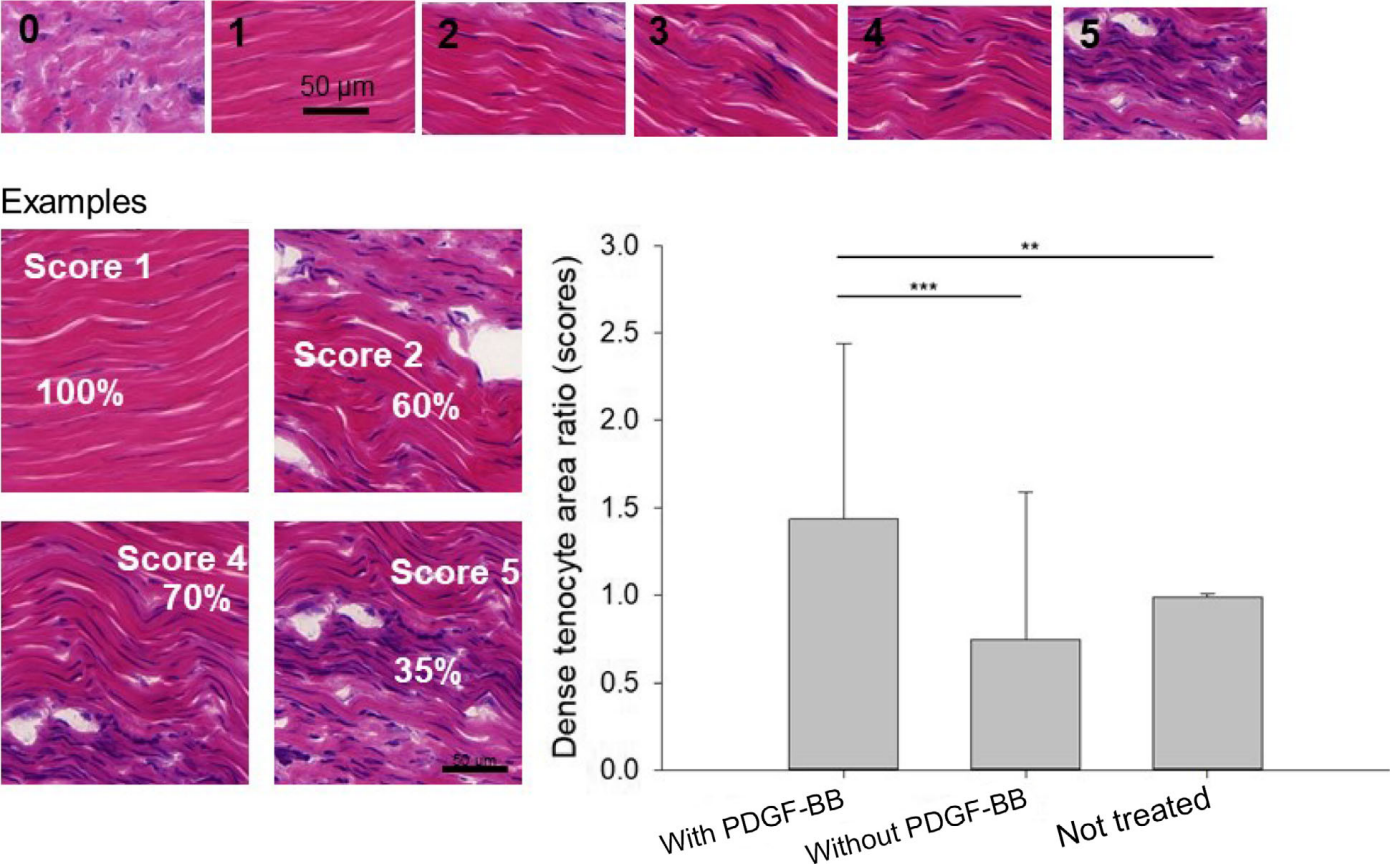
Dense tenocyte area ratio determined for PDGF‐BB release electrospun meshes 3 weeks postoperation. Semiquantitative scores were defined according to the density of tenocyte‐rich areas in cryogenic sections, stained with H&E. Additional images for scores 1–5 can be found in Fig. S3. Moreover, the percentage of these areas in 15 FOVs per tendon was determined. The ratio was calculated according to: ratio = score × %/100. For determination of the ratio, the zones with mainly mature tenocytes (with a slender spindle‐shaped morphology) have been considered. One‐way ANOVA was performed. *P *values < 0.05 were considered significant (*). If *P* < 0.01, this was marked by **, and if *P* < 0.001 by ***. Error bars indicate standard deviations. Biological independent replicates *n* = 6. Scale bars = 50 µm.

A significantly different dense tenocyte area ratio was found for tendons treated with or without PDGF‐BB (Fig. 2). The growth factor led to a significantly higher dense tenocyte area ratio, showing more cell‐rich zones with mature tenocytes, compared to DP‐treated tendons without PDGF‐BB and compared to NT tendons.

### Proteoglycan content

**Figure 3:**
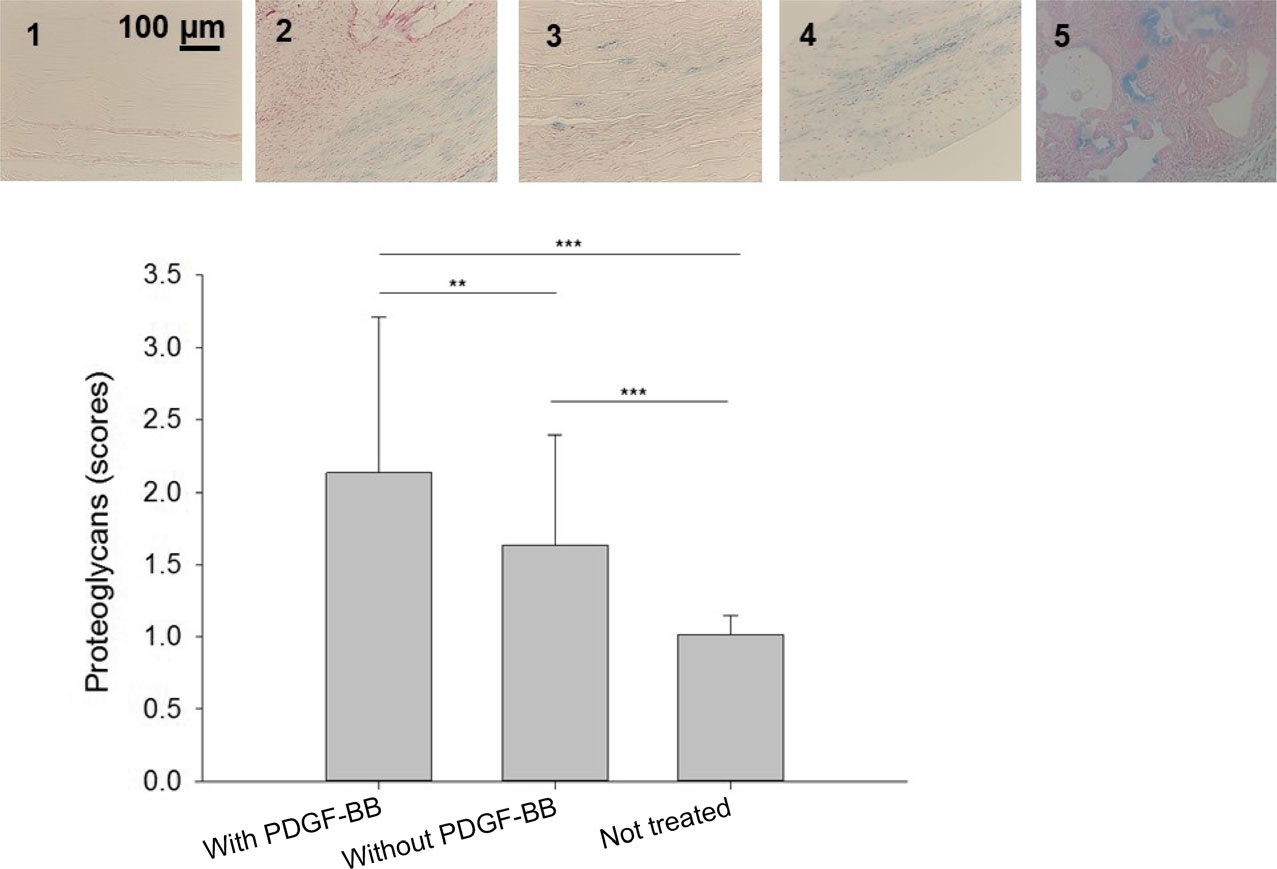
Proteoglycan content determined for PDGF‐BB release from full‐transection Achilles tendons: definition of scores by typical fields of view in Alcian blue‐stained paraffin sections (scores 1–5 above, scale bar = 100 µm) and semiquantitative determination (below). One‐way ANOVA was performed. *P* values < 0.05 were considered significant (*). If *P* < 0.01, this was marked by **, and if *P* < 0.001 by ***. Error bars indicate standard deviations. Biological independent replicates *n* = 6. Additional images for scores 1–5 can be found in Fig. S4.

Tendons that received a PDGF‐BB treatment showed a significantly higher proteoglycan content as assessed by Alcian blue staining (Fig. 3). Moreover, it was also significantly higher than in not treated tendons. In addition, tendons treated with tubes without PDGF‐BB had a significantly higher proteoglycan content compared to NT tendons.

### Alpha‐SMA

**Figure 4:**
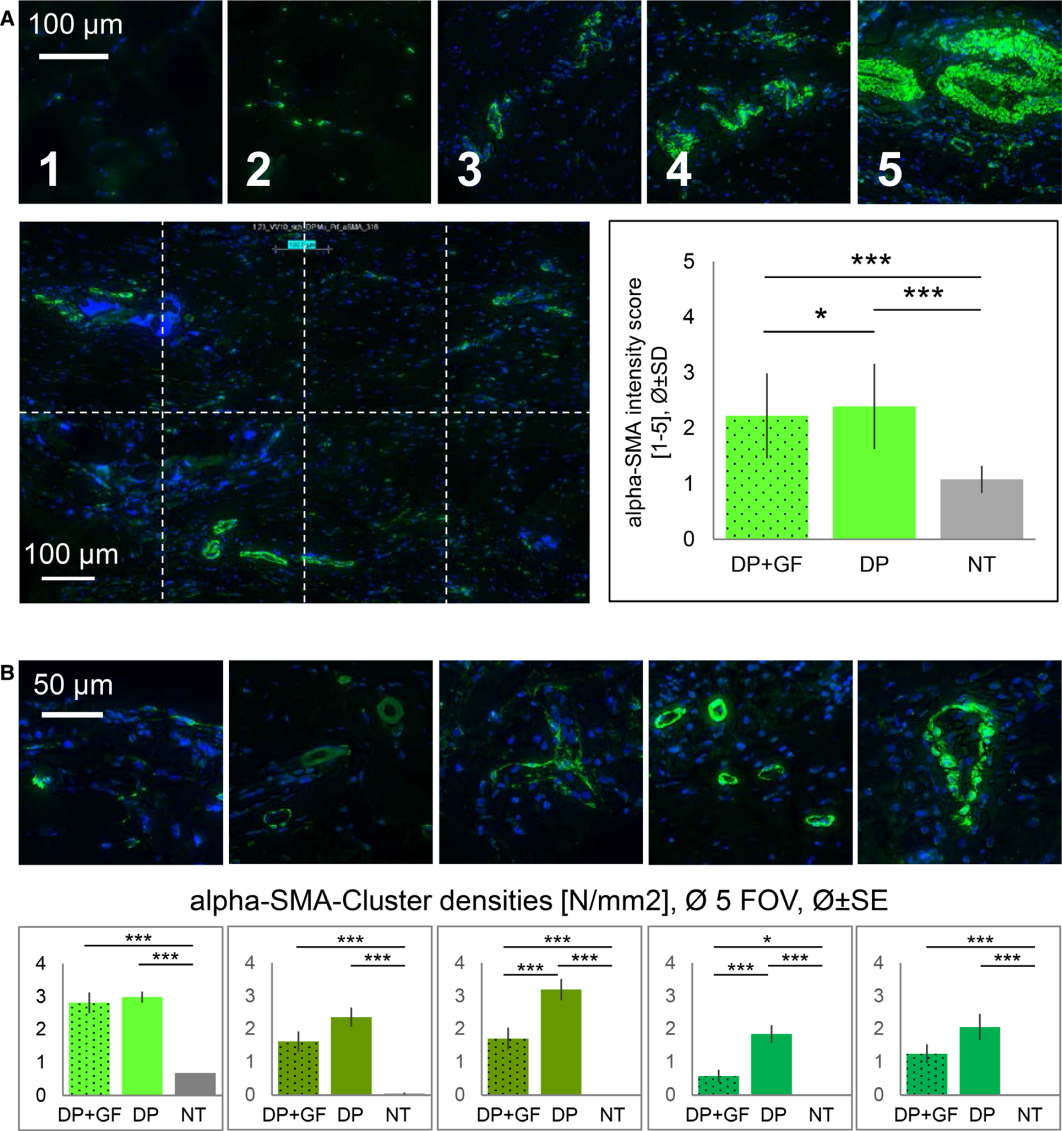
Alpha‐SMA^+^ cells. Semiquantitative scoring system: 1–5 definition (A top row), analysed in five FOVs, each FOV with eight rectangles (A bottom left); Cluster densities: visualization of cluster categories (B top row) and mean densities (B bottom row) of the corresponding cluster type. Cluster categories (from left to right): small full clusters with circumference < 100 µm; precursor vessels < 100 µm; precursor vessels > 100 µm; well‐developed vessels < 100 µm; well‐developed vessels > 100 µm. DP = DegraPol^®^, GF = PDGF‐BB, NT = not treated. One‐way ANOVA was performed. *P *values < 0.05 were considered significant (*). If *P* < 0.01, this was marked by **, and if *P* < 0.001 by ***. Error bars indicate standard deviations. Biological independent replicates *n* = 6. Scale bars in A = 100 µm; in B = 50 µm. Additional images for scores 1–5 can be found in Fig. S5A, and for different cluster size in Fig. S5B.

Alpha‐SMA expression was scored in immunohistochemically stained paraffin sections (Fig. 4). It was found that the delivery of PDGF‐BB to the wound site reduced alpha‐SMA expression significantly when compared to DP‐treated tendons without growth factor. Moreover, both tube‐treated tendons (±PDGF‐BB) had a significantly higher alpha‐SMA expression compared to NT tendons (Fig. 4A). Accordingly, the analysis of alpha‐SMA in the different frame cluster types revealed significantly lower densities in case of PDGF‐BB treatment compared to DP tube without PDGF‐BB (Fig. 4B); only the number of the smallest clusters, the precursor vessels < 100 µm and the well‐developed vessels > 100 µm did not differ between the treated groups; in addition, both groups showed always significantly higher alpha‐SMA clusters compared to not treated tendons.

### Collagens I and III

**Figure 5:**
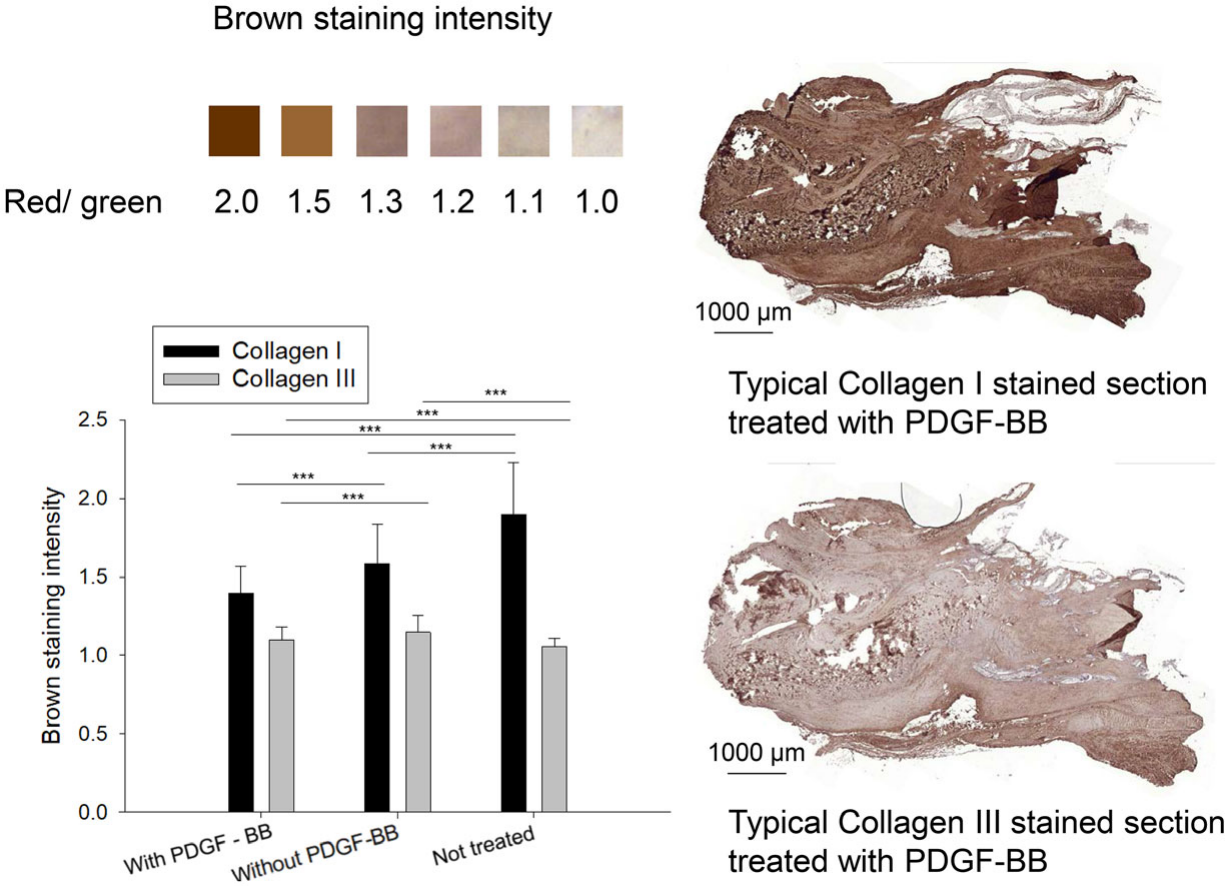
Collagen I and III immunohistochemistry of Achilles tendons in paraffin sections, with red/green ratio to assess brown staining intensity and typical examples for tendons stained with collagens I and III. One‐way ANOVA was performed. *P *values < 0.05 were considered significant (*). If *P* < 0.01, this was marked by **, and if *P* < 0.001 by ***. Error bars indicate standard deviations. Biological independent replicates *n* = 6. Scale bars = 1000 µm. Positive (NT) and negative (rabbit brain) controls for collagen I and III stainings are given in Fig. S6.

Collagens I and III were quantified in immunohistochemically stained longitudinal sections (Fig. 5). The brown intensity in not treated tendons was twice as intense in collagen I compared to collagen III. For both collagen I and collagen III, respectively, PDGF‐BB‐treated tendons showed lower brown intensities as assessed by the red/green ratio in the corresponding histograms. The presence of PDGF‐BB reduced the collagen I intensity to around 88% compared to PDGF‐BB‐free tendons; for collagen III, the corresponding value was 95%.

## Discussion

### Background

PDGF‐BB is a growth factor known to enhance mitogenesis, and it has been shown to support tendon healing 9. We therefore designed a polymer tube with incorporated PDGF‐BB 8 and assessed it in a full‐transection rabbit Achilles tendon model. In the present histomorphometric study, we examined the effects of PDGF‐BB that was released from the inner bioactive layer of a bilayered electrospun tube. Histomorphometry included analysis on a cellular and extracellular matrix level, considering cell migration and resulting distributions and proteoglycans, collagen types I and III and alpha‐SMA. The rather early time point postoperation (3 weeks) was chosen based on the release kinetics of PDGF‐BB from the polymer tube *in vitro* 8, which revealed more than 90% of the diffusible PDGF‐BB released at day 21.

### Main findings

PDGF‐BB promoted tendon healing by decreasing cell densities near the implant surface and near the endotenons, ending up in a more homogenous cell density at the repair site (Fig. 1). Moreover, PDGF‐BB‐treated specimen showed a significantly higher proteoglycan content (Fig. 3), while alpha‐SMA (Fig. 4), a marker of smooth muscle cells and myofibroblasts, and collagen III (Fig. 5), a transient type of collagen during tendon healing, were significantly lower when compared to tendons treated with tubes without PDGF‐BB.

### Proliferation and migration

It is well known that PDGF‐BB enhances proliferation of fibroblasts 17, 18, 19. In addition, PDGF‐BB acts via chemotaxis and has an influence on the cell migration. It has been shown that a porcine cross‐linked collagen sponge from Geistlich^®^ Pharma (Wolhusen, Switzerland) seeded with human gingival fibroblasts and either treated or not with PDGF‐BB increased the distance of the fibroblasts to the margin of the scaffold. In other words, PDGF‐BB enhanced the migration into the porous material and led to a more homogenous cell distribution within the sponge 20. Accordingly, we found that the density of tenocyte and tenoblast was significantly lowered directly next to the tube implant (Fig. 1F, zone Z4) and also near the endotenons (Fig. 1D, Z3 near EN). Correspondingly, the zones located further away from the implant and from the endotenon were populated with a significantly higher cell density (Fig. 1E, Z3 near DP). The cells apparently migrate from the vicinity of the implant and endotenons towards more distanced places, to sites between the PDGF‐BB secreting implant and the tenoblast‐rich endotenon. This results in an overall more homogenous cell distribution at the wound site. The healing process is therefore accelerated by PDGF‐BB: after a cellular and proliferative phase, cell density at the wound site declines to pave the way for the remodelling phase 21.

Nevertheless, we determined that dense tenocyte area ratios of mature tenocytes (and not tenoblasts) were found in higher numbers in the presence of PDGF‐BB compared to tendons treated without PDGF‐BB (Fig. 2). They were situated directly at the wound site where the proper tendon had been cut by a scalpel during surgery. To our knowledge, such clusters of mature tenocytes have not been reported previously. It might be speculated that they are formed under the influence of PDGF‐BB that, on the one hand, leads to more homogenous cell distribution of all types of cells and that, on the other hand, leads to an advanced maturation of the tenoblasts, resulting in clusters of mature tenocytes. From the maturity point of view, such tenocyte clusters attest an accelerated healing; from the cluster point of view, however, it rather puts proper healing into question.

### Proteoglycans

PDGF‐BB‐treated tendons showed significantly higher contents of proteoglycans compared to their PDGF‐BB‐free analogous tendons (Fig. 3). This was expected and stands in accordance with other studies. The PDGF‐BB has been shown to increase the proteoglycan content in rabbit tendons in a dose‐dependent manner; for intermediate and proximal intrasynovial flexor tendon segments, an effective concentration lied in the range of 0.1–30 and 0.1–100 ng·mL^−1^ PDGF‐BB, respectively 22. Up to 6 weeks postlaceration, glycosaminoglycan (GAG) content and accordingly proteoglycans are elevated in healing tendons 21. It may be speculated that a higher proteoglycan content at 3 weeks is attributed to an earlier GAG peak, meaning that the whole healing process has been accelerated and certain stages – here maximum proteoglycan content – reached at earlier time point postsurgery.

### Alpha‐SMA

In contrast to fetal tendons, the healing default pathway of adult tendons is the formation of fibrovascular scar 23. One central marker of fibrotic tissue and fibrosis in general is alpha‐SMA. Here, we clearly found that PDGF‐BB released from our tube implant reduced the alpha‐SMA^+^ cells in the healing Achilles tendons (Fig. 4). First, the general alpha‐SMA level in immunohistochemically stained sections was significantly lower; this included not only myofibroblasts 24, 25, a transient cell type during tendon healing, but also vascular smooth muscle cells 26. Second, the semiquantitatively scored alpha‐SMA^+^ clusters of various size, which are mainly attributed to vessels, were also significantly lower in the PDGF‐BB samples (except for the small clusters sized < 100 µm, which were similar, and the precursor vessels < 100 µm and the well‐developed vessels > 100 µm, which were lower, but not significantly lower). These findings are supported by a study from Comut *et al.* 27 who found a decreased alpha‐SMA protein synthesis in human gingival fibroblasts in the presence of 10 ng·mL^−1^ PDGF‐BB. Interestingly, the same study revealed a correlation between the fibroblast orientation and the alpha‐SMA synthesis of the cells, which we were, however, not able to detect in our experimental setting. In contrast, another research group found no effect of PDGF‐BB (10 ng·mL^−1^) on alpha‐SMA‐expressing cells in the torn human rotator cuff 28. Nevertheless, it was noted that alpha‐SMA‐containing cells could contribute adversely to the healing of the rotator cuff, by retraction of the torn ends.

A reduced alpha‐SMA protein amount in the presence of PDGF‐BB can be judged positively because it is attributed to less scar formation during the healing process. For example, hypertrophic scar fibroblasts co‐cultured with ASCs and receiving their secretome with many cytokines and trophic factors had a lower gene expression in alpha‐SMA compared to their monoculture 12. Although not primarily PDGF‐BB was applied to these hypertrophic scar fibroblasts, but a cocktail of many factors, this still supports our finding of lower alpha‐SMA expression as a positive, antifibrotic outcome. In addition, the lower densities of vessel‐like structures that were alpha‐SMA^+^ (smooth muscle cells) can be interpreted as a progression during tendon healing where angiogenesis and vascularization are mainly occurring immediately after the tendon rupture and decline over time 21. As such, lower vessel densities 3 weeks postlaceration imply an advanced state in the healing process.

### Collagen

The main type of collagen found in native healthy tendons is collagen I. During tendon healing, collagen type III is built up transiently and is finally replaced by collagen I at later stages 21. Here, we found that at 3 weeks postsurgery, the collagen III content was significantly lower in the case of PDGF‐BB treatment (Fig. 5). This might be associated with a faster tendon healing 29; in other words, the maximum of collagen III increase might have already been over during the healing, indicating a progressed stage.

We also found a lower collagen I content for PDGF‐BB‐treated tendons, standing in contrast to other PDGF‐BB studies where the growth factor stimulated collagen I production 30, 31, 32. Our findings of lower collagen I imply that other extracellular matrix (ECM) components – such as proteoglycans that were significantly increased by PDGF‐BB (Fig. 3) – reduced the relative amount of collagen I, ending up in a lower staining intensity.

### Limitations

Although we present a comprehensive histomorphometric analysis of the healing tendon tissue in the presence of a sustained PDGF‐BB delivery from an electrospun tube, there are several limitations to this study. First, biomechanical properties of the tendons would be interesting to assess. Second, longer time points postsurgery, but also shorter time points than 3 weeks would help to elucidate the mechanism of PDGF‐BB. Last, adhesion extent of the healing tendon to the surrounding tissue in the presence and absence of PDGF‐BB would be a further interesting topic to address. For this, sample processing would be different than in this study presented here, with more cross‐sectional sections rather than longitudinal ones.

## Summary and conclusion

The sustained delivery of PDGF‐BB to a fully transected Achilles tendon had positive effects with respect to the cellular and ECM healing pattern. It reduced fibrotic markers such as alpha‐SMA, accelerated the healing as indicated by a lower collagen III intensity and finally led to a more homogenously distributed cellular density. Thus, providing PDGF‐BB in a controlled sustained manner to the wound site is promising with regard to tendon rupture repair and might find its way into clinical practice.

## Conflict of interest

The authors declare no conflict of interest.

## Author contributions

GMB processed tissue samples for histology, performed immunohistological staining and imaging with slide scanner, analysed all histological sections, made Fig. 4 and edited the manuscript. OE fabricated electrospun tubes with or without PDGF‐BB, processed tissue samples for histology, performed immunohistological staining and imaging with slide scanner and edited the manuscript. MC operated all rabbits and supervised the study. CS synthesized the polymer used for the tubes. PG supervised the study. JB designed experiments, made Figs 1, 2, 3 and 5, performed statistical analyses and wrote the manuscript.

## Supporting information


**Fig. S1.** Overview of typical longitudinal sections. A tendon that received a DP tube after surgical repair. Magnification: 8×. Haemalaun Sudan staining (left) and Picrosirius Red staining (right). Areas A and B (the small green rectangles) are magnified below.
**Fig. S2.** Macroscopic images. Macroscopic images of the healing tendons three weeks postsurgery. Based on NT (no treatment) tendons as reference, the cross‐sectional area of the treated tendons increased; it was 248 ± 59% (tube without PDGF‐BB) and 211 ± 62% (tube with PDGF‐BB) relative to NT (100 ± 18%). The length of the treated tendons also increased compared to NTs: 135 ± 11% (tube without PDGF‐BB) and 110 ± 20% (tube with PDGF‐BB) relative to NT (100 ± 19%).
**Fig. S3.** Scoring system for tenocyte rich areas. Examples for semiquantitative scores with different densities of tenocyte rich areas. This is additional information to Figure 2 in the main manuscript.
**Fig. S4.** Scoring system for proteoglycans. Examples for semiquantitative scores with different Alcian blue intensities referring to different proteoglycan content. This is additional information to Figure 3 in the main manuscript.
**Fig. S5.** Scoring system for alpha‐SMA. Representative images for semiquantitative scores with different amounts of alpha‐SMA^+^ cells (A). Examples for clusters (B) with categories (from top to down row): small full clusters with circumference < 100 µm; precursor vessels < 100 µm; precursor vessels > 100 µm; well developed vessels < 100 µm; well developed vessels > 100 µm. This is additional information to Figure 4A and 4B, respectively, in the main manuscript.
**Fig. S6.** Positive and negative controls for collagen I and collagen III staining. Native rabbit tendons were used as positive control (top) and native rabbit brain tissue was used as negative control.
