# Supplementary material for: Impact of PDGF‐BB on cellular distribution and extracellular matrix in the healing rabbit Achilles tendon three weeks post‐operation
Source: FEBS Open Bio. 2020 Feb 5;10(3):327–37. doi: 10.1002/2211-5463.12736 (PMC7050259; doi:10.1002/2211-5463.12736)
Supplement: Supplementary file 1 — Fig. S1. Overview of typical longitudinal sections. A tendon that received a DP tube after surgical repair. Magnification: 8×. Haemalaun Sudan staining (left) and Picrosirius Red staining (right). Areas A and B (the small green rectangles) are magnified below. Fig. S2. Macroscopic images. Macroscopic images of the healing tendons three weeks postsurgery. Based on NT (no treatment) tendons as reference, the cross‐sectional area of the treated tendons increased; it was 248 ± 59% (tube without PDGF‐BB) and 211 ± 62% (tube with PDGF‐BB) relative to NT (100 ± 18%). The length of the treated tendons also increased compared to NTs: 135 ± 11% (tube without PDGF‐BB) and 110 ± 20% (tube with PDGF‐BB) relative to NT (100 ± 19%). Fig. S3. Scoring system for tenocyte rich areas. Examples for semiquantitative scores with different densities of tenocyte rich areas. This is additional information to Figure 2 in the main manuscript. Fig. S4. Scoring system for proteoglycans. Examples for semiquantitative scores with different Alcian blue intensities referring to different proteoglycan content. This is additional information to Figure 3 in the main manuscript. Fig. S5. Scoring system for alpha‐SMA. Representative images for semiquantitative scores with different amounts of alpha‐SMA+ cells (A). Examples for clusters (B) with categories (from top to down row): small full clusters with circumference < 100 µm; precursor vessels < 100 µm; precursor vessels > 100 µm; well developed vessels < 100 µm; well developed vessels > 100 µm. This is additional information to Figure 4A and 4B, respectively, in the main manuscript. Fig. S6. Positive and negative controls for collagen I and collagen III staining. Native rabbit tendons were used as positive control (top) and native rabbit brain tissue was used as negative control. [file FEB4-10-327-s001.docx]

Supporting Information

**Impact of PDGF-BB on cellular distribution and extracellular matrix in the healing rabbit Achilles tendon three weeks post-operation**

Gabriella Meier Bürgisser^1^, Olivera Evrova, Ph.D.^1,2^, Maurizio Calcagni, M.D.^1^, Chiara Scalera^4^, Pietro Giovanoli, M.D.^1^ and Johanna Buschmann, Ph.D.^1^*

^1^Division of Plastic Surgery and Hand Surgery, University Hospital Zurich, Sternwartstrasse 14, 8091 Zurich, Switzerland

^2^Laboratory of Applied Mechanobiology, ETH Zürich, Vladimir-Prelog-Weg 1-5/ 10, 8093 Zurich, Switzerland

^3^ab medica, via J. F. Kennedy, 10/12 - 20023 Cerro Maggiore (MI), Italy

* Corresponding author:

*PD Dr. Johanna Buschmann*

University Hospital Zurich, ZKF, Division of Plastic Surgery and Hand Surgery

Sternwartstrasse 14, 8091 Zurich, Switzerland

Phone: +41 44 255 98 95

Fax: +41 44 255 50 47

e-Mail: [johanna.buschmann@usz.ch](mailto:johanna.buschmann@usz.ch)

**Short Title**: PDGF-BB release to tendon rupture repair model

**Word count**: 213 (abstract); 3559 (main text); 5 figures and 6 supporting information figures

**Keywords:** Rabbit Achilles tendon; alpha-SMA, collagen, PDGF-BB, proteoglycan

Submitted to

***FEBS Open Bio***

**
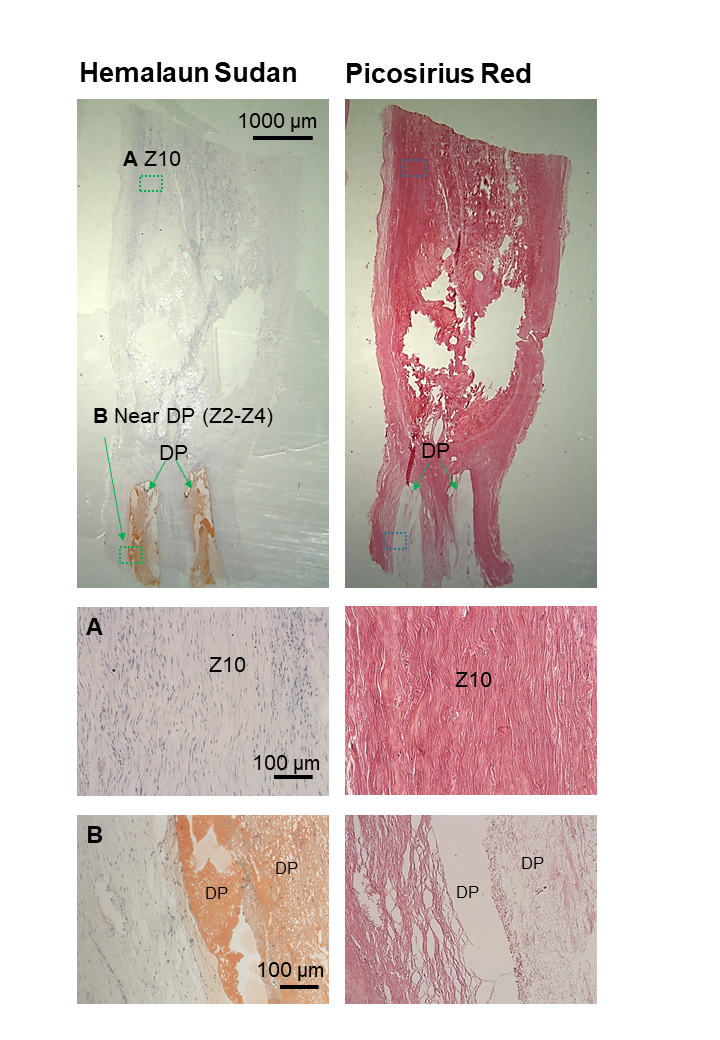
**

**SI Figure 1** **Overview of typical longitudinal sections**. A tendon that received a DP tube after surgical repair. Magnification: 8x. Haemalaun Sudan staining (left) and Picosirius Red staining (right). Areas **A** and **B** (the small green rectangles) are magnified below.


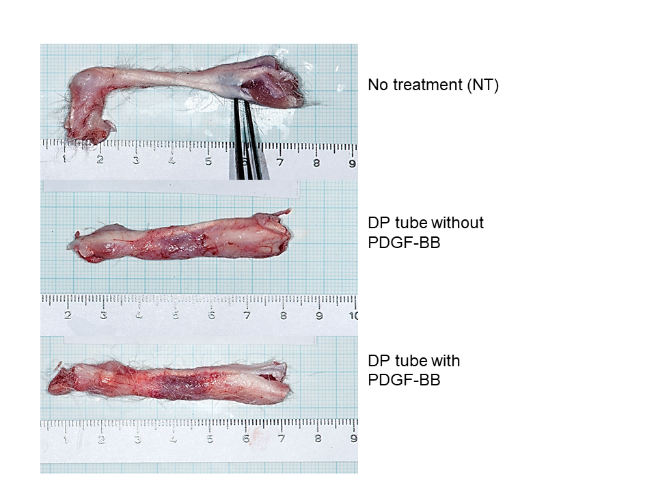


**SI Figure 2** **Macroscopic images**. Macroscopic images of the healing tendons three weeks post-surgery. Based on NT (no treatment) tendons as reference, the cross-sectional area of the treated tendons increased; it was 248 ± 59 % (tube without PDGF-BB) and 211 ± 62 % (tube with PDGF-BB) relative to NT (100 ± 18 %). The length of the treated tendons also increased compared to NTs: 135 ± 11 % (tube without PDGF-BB) and 110 ± 20 % (tube with PDGF-BB) relative to NT (100 ± 19 %).


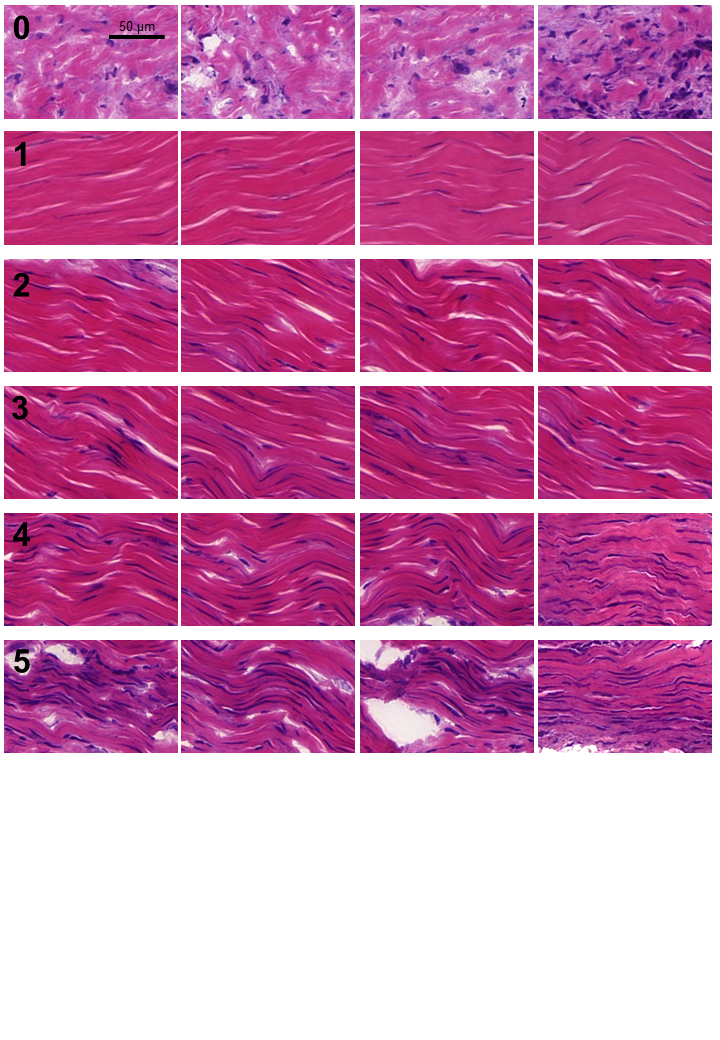


**SI Figure 3** **Scoring system for tenocyte rich areas**. Examples for semi-quantitative scores with different densities of tenocyte rich areas. This is additional information to Figure 2 in the main manuscript.


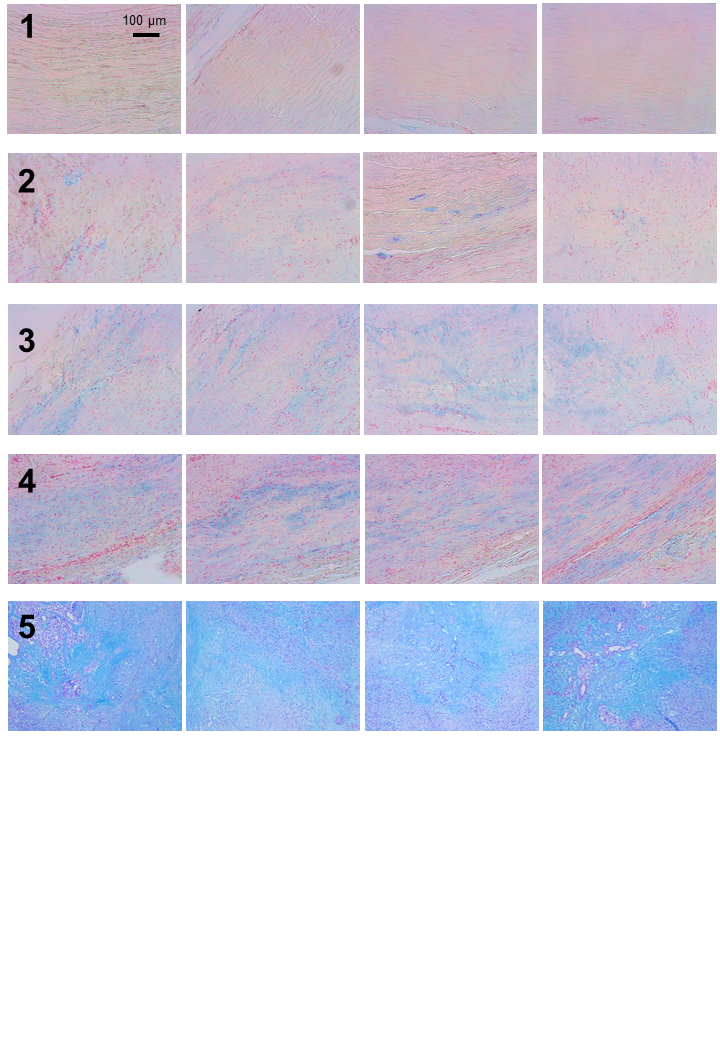


**SI Figure 4** **Scoring system for proteoglycans**. Examples for semi-quantitative scores with different Alcian blue intensities referring to different proteoglycan content. This is additional information to Figure 3 in the main manuscript.


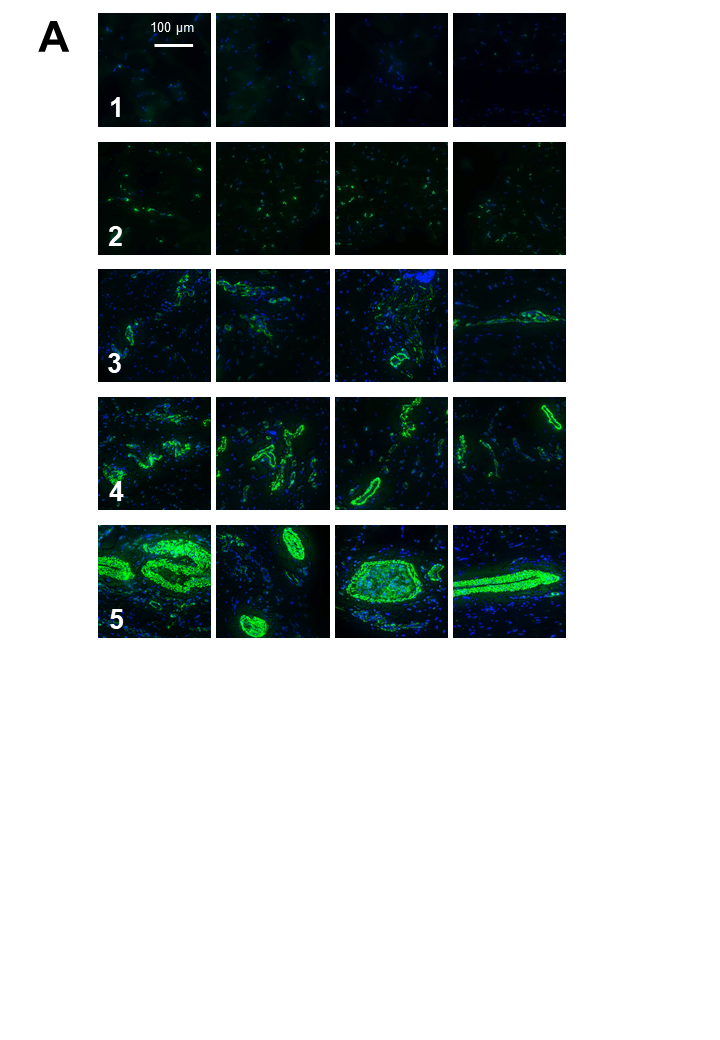

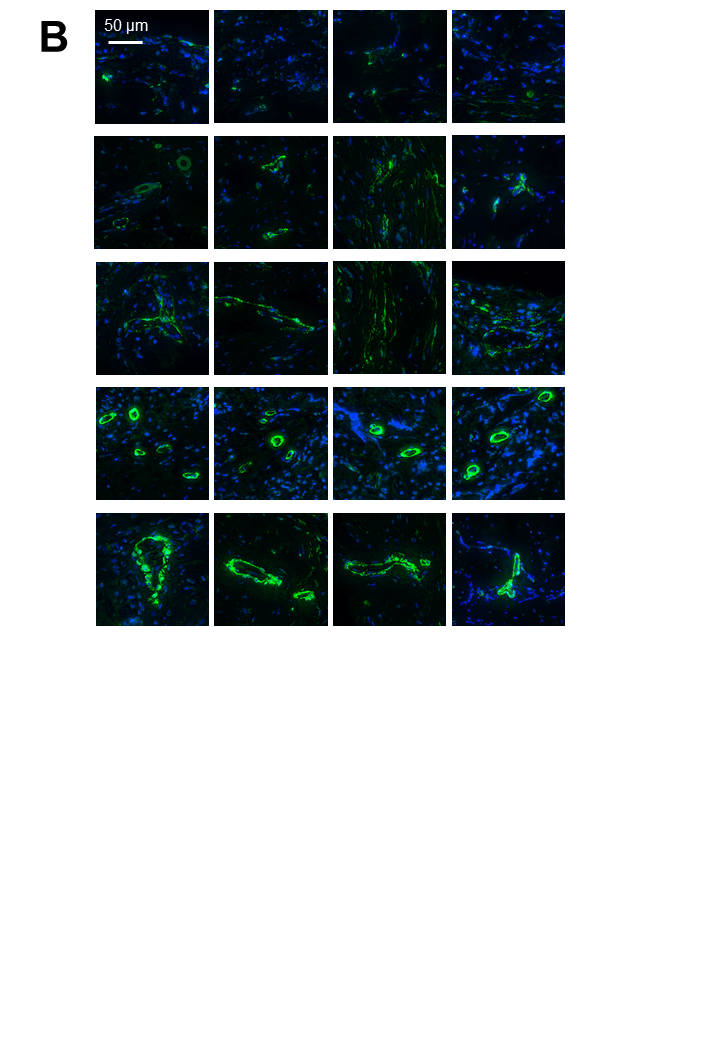


**SI Figure 5** **Scoring system for alpha-SMA.** Representative images for semi-quantitative scores with different amounts of alpha-SMA^+^ cells (A). Examples for clusters (B) with categories (from top to down row): small full clusters with circumference < 100 µm; precursor vessels < 100 µm; precursor vessels > 100 µm; well developed vessels < 100 µm; well developed vessels > 100 µm. This is additional information to Figure 4A and 4B, respectively, in the main manuscript.


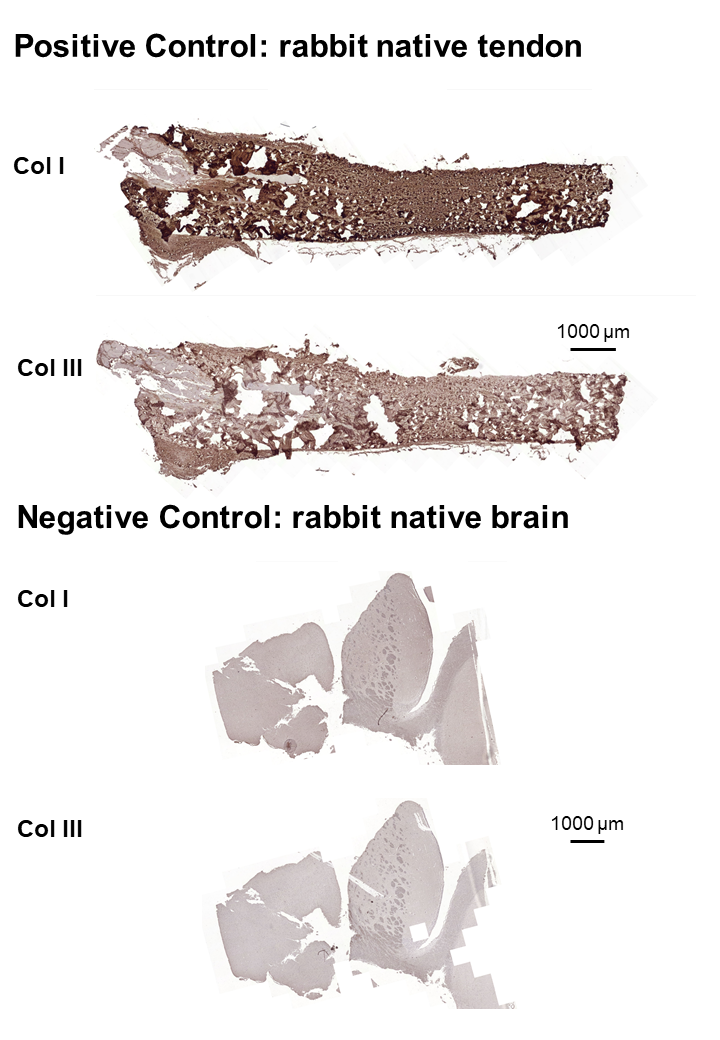


**SI Figure 6** **Positive and negative controls** **for collagen I and collagen III staining.** Native rabbit tendons were used as positive control (top) and native rabbit brain tissue was used as negative control.
